# Role of Tunneling Nanotubes in Viral Infection, Neurodegenerative Disease, and Cancer

**DOI:** 10.3389/fimmu.2021.680891

**Published:** 2021-06-14

**Authors:** Vaibhav Tiwari, Raghuram Koganti, Greer Russell, Ananya Sharma, Deepak Shukla

**Affiliations:** ^1^ Department of Microbiology & Immunology, Chicago College of Osteopathic Medicine, Midwestern University, Downers Grove, IL, United States; ^2^ Department of Ophthalmology and Visual Sciences, University of Illinois at Chicago, Chicago, IL, United States; ^3^ Department of Biomedical Sciences, College of Graduate Studies, Midwestern University, Downers Grove, IL, United States; ^4^ Vanderbilt University School of Medicine, Nashville, TN, United States; ^5^ Department of Microbiology and Immunology, University of Illinois at Chicago, Chicago, IL, United States

**Keywords:** tunneling nanotubes (TNT), heparan sulfate, virus cell interactions, virus surfing, virus cell to cell spread

## Abstract

The network of tunneling nanotubes (TNTs) represents the filamentous (F)-actin rich tubular structure which is connected to the cytoplasm of the adjacent and or distant cells to mediate efficient cell-to-cell communication. They are long cytoplasmic bridges with an extraordinary ability to perform diverse array of function ranging from maintaining cellular physiology and cell survival to promoting immune surveillance. Ironically, TNTs are now widely documented to promote the spread of various pathogens including viruses either during early or late phase of their lifecycle. In addition, TNTs have also been associated with multiple pathologies in a complex multicellular environment. While the recent work from multiple laboratories has elucidated the role of TNTs in cellular communication and maintenance of homeostasis, this review focuses on their exploitation by the diverse group of viruses such as retroviruses, herpesviruses, influenza A, human metapneumovirus and SARS CoV-2 to promote viral entry, virus trafficking and cell-to-cell spread. The later process may aggravate disease severity and the associated complications due to widespread dissemination of the viruses to multiple organ system as observed in current coronavirus disease 2019 (COVID-19) patients. In addition, the TNT-mediated intracellular spread can be protective to the viruses from the circulating immune surveillance and possible neutralization activity present in the extracellular matrix. This review further highlights the relevance of TNTs in ocular and cardiac tissues including neurodegenerative diseases, chemotherapeutic resistance, and cancer pathogenesis. Taken together, we suggest that effective therapies should consider precise targeting of TNTs in several diseases including virus infections.

## Introduction

The ability of a cell to communicate with the distant neighboring cell is vital for its survival and efficient function. Many pharmaceuticals work by enhancing or inhibiting components of the cell communication mechanisms (1, 2). The formation of filamentous (F)-actin rich tubules termed tunneling nanotubes (TNTs) are one such mechanism of communication between the cells (3, 4). TNTs have been broadly defined as thin membrane tubes which connect two cells and mediate the transfer of cellular cargo (5, 6). A recent review has added more detail to this definition, stating that TNTs must satisfy three requirements: they must connect two or more cells, be composed of F-actin, and not come in contact with the substrates that pass through them (7). In this review, we use the more specific definition to differentiate TNTs from similar but distinct structures.

TNTs exist as a cytoplasmic bridge between the two closely or distant cells. They form gap-like junctions between connected cells and mediate the exchange of cytoplasmic proteins, cellular organelles (such as endoplasmic reticulum, Golgi, endosome, lysosome, mitochondria), lipids, nucleic acids, microRNA, ions, calcium, and several other components (4, 8–10). The diameter of the TNT restricts the type of cargo that can be transported. Thinner TNTs contain F-actin and are less than 7 µm in diameter which prevents them from transferring large organelles between cells (11). Thicker TNTs contain microtubules and exceed a 7 µm diameter which allows them to transfer organelles such as mitochondria between cells (6, 12). They facilitate both short and long-distance direct communication, spanning distances of up to 300 µm (13). TNTs are able to polymerize and depolymerize rapidly in 30-60 seconds, making them fluid, transient structures (8, 14). TNTs have been shown to be formed by different cell types including epithelial and fibroblasts (15–17), neuronal (18, 19), and multiple types of immune cells (20).

**Figure 1:**
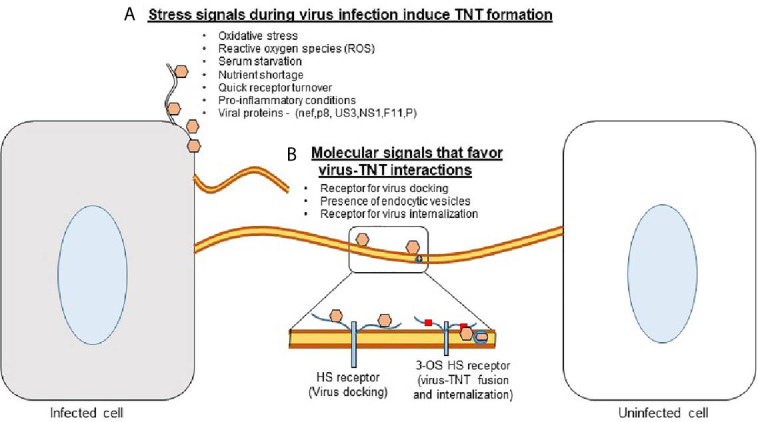
Tunneling nanotubes (TNTs) - a major platform for the viruses to scan the neighborhood for successful entry and spread. **(A)** Stress conditions (ROS, a lack of nutrients, and possibly a quick receptor turnover) together with the influx of viruses induce the Rho GTPase-mediated signaling pathways which then trigger the induction of TNTs. **(B)** The presence of viral receptor such as heparan sulfate (HS) provides an opportunity for the virion to dock and/or surf on TNTs. In addition, the presence of 3-O sulfated HS receptor may induce direct virus-TNT fusion or receptor mediated endocytosis to promote virus internalization.

While numerous processes are downregulated when cellular supplies are low and cells are under distress, TNT polymerization is enhanced (21). Cellular stress such as viral infections, damage by UV light, or hydrogen peroxide-mediated oxidation have all been shown to upregulate TNT formation (**Figure 1**) (22). TNT formation has recently been shown to play a large role in the pathogenesis of many diseases (23). Viral particles, prions, fungal spores, organelles, and other molecules can be transported by TNTs (23, 24). The concomitant evolution of pathogens with the cell’s ability to produce TNTs is interesting from a cellular biology standpoint and highlights the importance of targeting this mechanism of propagation to treat certain diseases.

## TNTs *Versus* Filopodia

Cells utilize numerous mechanisms for communication and sensing their environment such as filopodia. Filopodia, like TNTs, can form long range intercellular bridges (25). However, TNT structures are direct cytoplasm-to-cytoplasm bridges and can be much larger than the ~2.37-5.8 uM of filopodia (3, 26). Viruses, such as human immunodeficiency virus (HIV), can induce the formation of filopodia in dendritic cells and use them to surf onto connected naive cells (6, 27, 28). It was previously hypothesized that TNTs are a subsect of filopodia; but recent evidence suggests otherwise. For example, TNTs are free-floating while filopodia are connected to the cell plate. Unlike TNTs, filopodia are unable to mediate vesicular transport. Furthermore, the regulatory mechanisms to produce filopodia and TNTs work in direct opposition (29). In fact, when a cell encounters a TNT, it retracts its existing filopodia (29). Differential signaling pathways involved in TNT and filopodia formation are discussed below. Overall, the evidence suggests that while filopodia and TNTs are similar in structure, they are in fact separate entities.

## Signaling Formation of TNTs

Numerous studies have attempted to elucidate the signaling cascade involved in the formation of TNTs. Delage et al., 2016 reported that CDC42, IRSp53, and VASP form a complex which inhibits TNT formation and vesicular transport (29). Interestingly, these cell-signaling molecules have been shown to promote filopodia formation (30, 31). Conversely, Eps8 overexpression was shown to increase the extent of TNT connections and vesicular transport. Hypothesized mechanisms for this phenomenon suggest that Eps8 works synergistically with IRSp53 to bundle actin (32). Thus, IRSp53 plays a major regulatory role in inducing or inhibiting TNT formation. Furthermore, it shows how the formation of filopodia and TNTs can utilize opposing signaling routes.

As TNTs form in response to cellular stress, the molecules involved in DNA repair and cell cycle arrest could be involved in the upregulation of TNTs. p53-activated pathways were reported to be essential for production of TNTs (33). Cells induce TNT formation first by activating p53 which subsequently upregulates epidermal growth factor (EGF). EGF activates the Akt/PI3k/mTOR pathway to induce the actin polymerization necessary for TNT production (33). This process is likely mediated through M-sec, a protein known to affect the cytoskeleton that was discovered when examining microfold or M-cells of the intestine (16). Upregulation of M-sec induced the formation of TNTs, and knock-out M-sec models decreased TNT formation by as much as one third (16). M-sec has been shown to work with Ras associated small GTPase RalA which has been shown to be involved with actin cytoskeletal rearrangement (16, 34). Since TNTs are composed of mainly F-actin, the RalA-M-sec interaction may mediate TNT formation.

Recent work has explored the mechanisms behind TNT formation in neuronal cells. Overexpression of Rab35, a small GTPase involved in membrane recycling, induces TNT formation while inhibition of TNTs occurs with the addition of a dominant negative mutant of Rab35 (35). ACAP2, a downstream effector of Rab35, was shown to positively regulate TNT formation using a similar approach (35). ACAP2 does so through the inhibition of ARF6 which allows PI4P to recruit EHD1 (35). Loss of EHD1 reduces TNT production, suggesting a linear pathway by which Rab35-ACAP2-ARF6-EHD1 regulate TNTs in neuronal cells (35). In addition to the Rab35 pathway, Wnt signaling initiates TNT formation in neuronal cells. The addition of Wnt7a to CAD cells stimulates TNT production *via* the Wnt/Ca^2+^ pathway (36). CAD cells have been demonstrated to transfer α-syn fibrils using Wnt-induced TNTs (36).

Additionally, a 2017 study elucidates the importance of focal adhesion kinase (FAK) in the formation of TNT in squamous cancer cells *via* the upregulation of the MPP-2 metalloprotease (37). While MAPK pathways or microtubule inhibitors were shown to play a role in TNT formation, IP3 pathways and actin polymerization were reported to induce TNT formation (37).

**Figure 2:**
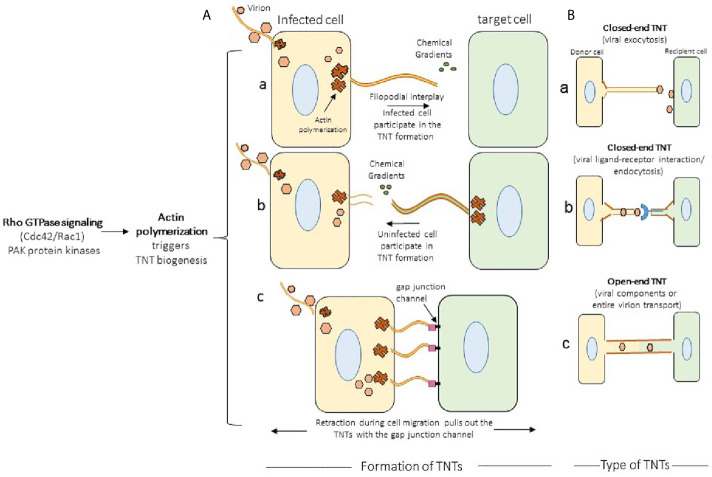
Mechanism of TNTs formation. **(A)** Actin polymerization together with Rho GTPase signaling leads to the formation of TNT bridges in which either infected cell (pane a; example, herpes simplex virus) or an uninfected cell (panel b; murine leukemia virus) forms the TNTs. The well characterized TNT biogenesis model in HIV and pseudo rabies virus (PRV) demonstrate the transient expression of cell adhesion molecules stabilizes the TNTs. The bridges of TNTs are also formed when the two connected cells retract during their migration resulting the formation of TNTs (panel c). **(B**) The types of TNTs. In closed-end TNTs, the cytoplasmic mixing between the donor and the target cells are not achieved, while in the open-end TNTs displays cytoplasmic continuity. Both type of TNTs aid in the viral spread, but it has been suggested that open-ended TNT leads to the generation of multinucleated giant cells.

**Figure 3:**
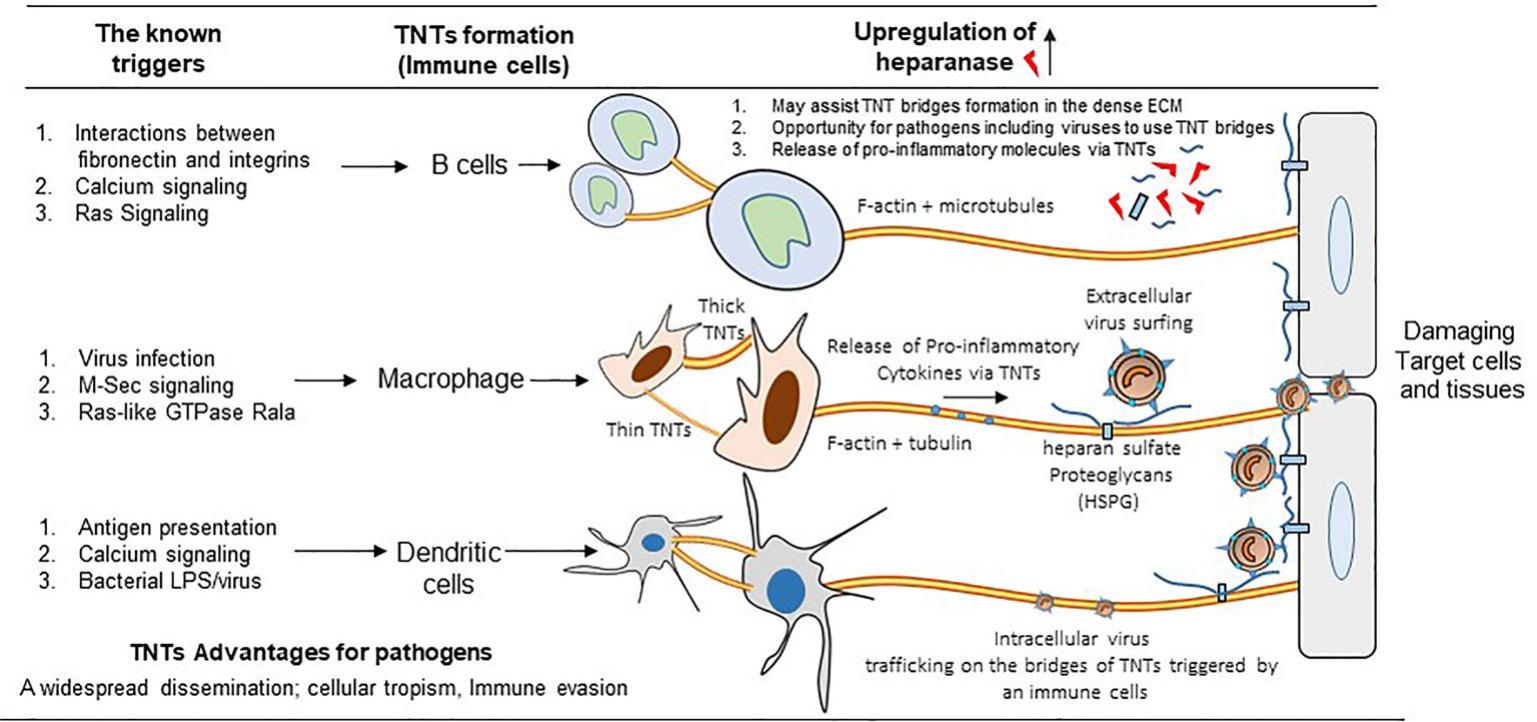
Significance of TNTs among the circulating immune cells. The cartoon highlights the common triggers including the virus infection generating various types of TNTs among B cells, macrophages, and dendritic cells. In parallel, the upregulation of heparanase together with the presence of heparan sulfate and the release of pro-inflammatory cytokines further aids in the TNT dependent viral infectivity (either the extracellular and or the intracellular virus trafficking using the circulating immune cells) resulting widespread dissemination of the virus to affect distantly located multiple organs.

In general, the TNTs formation are preferentially driven by the activation of Rho GTPase signaling with the polymerization of the filamentous actin at the cellular tip (14, 38). The actin rich filopodia-like protrusions make the bridges of TNTs by contacting the target cells. In the proposed model the filopodia from the viral infected cells starts forming TNTs (**Figure 2A**, panel a) (39). In contrast, in case of murine leukemia virus it is the uninfected cell that initiates the TNT formation (**Figure 2A**, panel b) (17). In the literature TNT can also be formed when a cell that is adhered to another cell starts moving away leaving the retracted actin rich TNTs between the cells (**Figure 2A**, panel c) (40). Using time-lapse microscopy both of the above mechanisms of TNT formation have also been reported in context with immune cells. For example, F-actin polymerization driven process is observed in dendritic cells, when the filopodia of two retracting cells turn into TNTs to maintain contact between the two cells. Interestingly, macrophages, are reported to use either of these mechanisms to form TNTs (40). Depending on the cytoplasmic connectivity between the TNT either from the donor and or recipient cell, they are further classified as closed-end and or open-end TNTs (**Figure 2B**, panels a-c) (6). Current model further suggests that actin containing TNT are shorter and thinner compared to TNTs containing tubulin alone or together with actin. The later type of TNTs rich in both actin and tubulin are widely documented (39). The ability of a given cell to form only one type or from multiple other types of TNTs remains unknown. In addition, the mechanism and the triggers are uniquely associated with the given TNTs are the same or they vary depending on cell type (38). However, the associated triggers such as heparanase upregulation, hypoxia, calcium signaling, ROS during inflammation may have profound effect on TNT biogenesis in the circulating immune cells, which in tuns may fastens the dissemination of the virus to affect multiple organs and contribute towards the associated pathologies (**Figure 3**). Interestingly, the properties of TNTs can vary from cell to cell. For instance, TNTs in T cells have very low permeability to calcium and contain distinct membrane linkages when compared to TNTs in macrophages (12, 41). Additionally, TNT produced in SCC cells are 2 times thicker and 2.5 times larger in length then those of PC12 cells (37). Furthermore, the SCC TNTs contain microtubules in contrast PC12 TNTs which only contain F-actin.

## Role of TNTs in Ocular Tissues

Previous studies have shown that TNTs are quite common in both anterior and posterior ocular cells such as corneal, trabecular meshwork, and retinal epithelial cells (10). It is believed that widely spaced cells in cornea communicate using TNTs (42). Confocal imaging has also provided unique evidence that TNT mediates connection between the inflammatory macrophages (43). It has also been suggested that TNT plays a rescue role in improving ocular pathologies such as and elevated intraocular pressure (IOP) (10, 44). For instance, macrophage mediate the transfer of cystinosin lysosomes from healthy donor cells to diseased cells *via* TNTs (44). The study lays the foundation that TNTs may serve as a tool to deliver the drug during transplantation with healthy donor cells in the cornea. Similarly, blockage of trabecular meshwork (TM) cells results in a higher IOP - a risk factor for glaucoma. In this regard, it has been shown that TM cells communicate *via* TNTs as a sensing mechanism during changes in IOP and cellular remodeling to allow additional aqueous fluid to normalize the eye pressure. In support, it has been shown that TM cells in glaucoma have prolonged TNT connections compared to normal TM cells (45). In retinal cells, the use of TNTs has been implicated in the transfer of the mitochondria.

In contrast, ocular inflammation either during localized acute and or chronic systemic origin are known triggers for TNTs due to exposure to growth factors (43). It has been suggested that TNTs could be exploited *in vivo* to deliver immunomodulatory protein which could either inhibit or promote inflammation. *In vitro* studies provide the evidence for the presence of tenascin-C in TNTs which are indication for their critical role in tissue remodeling (46).

## TNT Connections in Cardiac Tissues

Ischemic damage to cardiac tissue following myocardial infarction lends to the pathophysiology and development of heart failure. With heart failure ranking as a top contributor of cardiac death in industrialized nations, research exploring the regenerative potential of cardiomyocytes has become critical in understanding post-infarction recovery of cardiac tissue (47). Though cardiac tissue itself has limited regenerative capacity, studies have demonstrated that mesenchymal stem cells (MSCs) potentiate cardiomyocyte regeneration through cell-to-cell communication that includes paracrine signaling pathways and nanotubular connections (47). Utilizing co-culture models of rat cardiomyocytes, Figeac et al., 2014 explored the interaction between TNT-mediated communication processes and paracrine signaling of MSCs in response to cardiomyocyte (CM) injury. The researchers identified cross-talk between the two routes of communication, with TNTs mediating interaction between damaged CMs and human multipotent adipose derived stem cells (hMADS) and also altering the MSC secretome to influence the release of angiogenic growth factors, pro-inflammatory cytokines, and additional recruitment of bone marrow-derived MSCs (47). Other co-culture studies have shown the ability of nanotubular structures to directly facilitate differentiation of endothelial progenitor stem cells into cardiomyocytes (48). Mitochondria and cytoplasmic GFP travel along nanotubular tracts bridging neonatal rat CMs and undifferentiated adult human endothelial progenitor cells (EPCs) (48). This unidirectional transfer of macromolecular complexes was followed by differentiation of EPCs *in vitro* (48). In conditions of ischemic injury, Cselenyák et al., 2010 also demonstrated TNT-mediated mitochondrial transfer (49). Creating an *in vitro* ischemic model using H9c2 cardiomyoblasts, these researchers showed that nanotubular connections (200-500 nm wide) between MSCs and injured cardiomyoblasts functioned to facilitate organelle transfer, resulting in rescue of damaged cells (49). While TNTs have been implicated in connecting MSCs and CMs and facilitating unidirectional exchange of macromolecules and organelles, nanotubular tracts have also been discovered between major cardiac cell types as a means of long distance cell-to-cell communication (50). It is thought that such structures enable bidirectional mitochondrial exchange and propagation of electrical potential *via* Ca2+ signaling (50).

In addition to their role in potentiating regeneration and rescue of cardiac tissue, TNTs have also been identified as players in the electrical signaling pathway of the heart (50, 51). While CMs have been regarded as the primary cells responsible for producing and propagating action potentials, nanotubular connections and connexins mediate passive electrical activity within nonmyocytes (51). Utilizing an optogenetic mice model, researchers indicated that TNTs played a potential role in bridging both cell types and allowing for electronic coupling in areas of scarring, where there are abundant myocytes and nonmyocytes present (51).

Overall, TNTs play critical roles in cell-to-cell communication in cardiac tissue. Further research delineating the mechanisms of mitochondrial transfer, re-framing current understanding of cardiac electrical connectivity, and *in vivo* experimentation is necessary to gain an appreciation for the role of TNTs in cardiovascular pathophysiology. A thorough understanding of nanotubular connections poses an avenue to develop potential therapies for heart failure and ischemic damage.

## TNTs in Viral Infection

**Figure 4:**
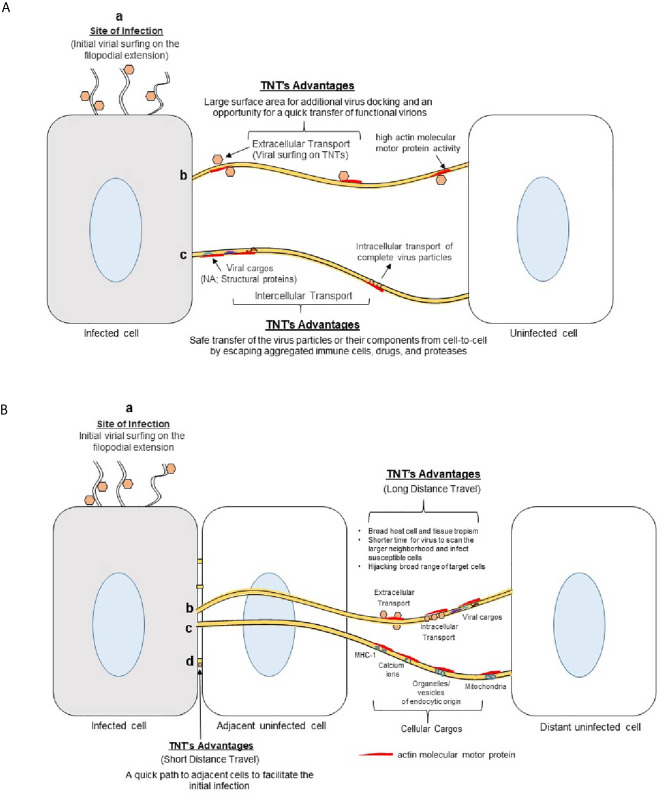
Advantages of using TNTs bridges in viral spread. **(A)** Viruses use actin-rich filopodia to surf to the cell surface. This surfing movement helps the viruses to dock and concentrate at the target cell’s surface to facilitate viral attachment and entry. (b) Viruses also surf using TNT bridges to move extracellularly between cells during early phases of infection. The larger surface area provided by the TNTs allows for increased viral docking - an opportunity for quick viral transfer to the nearby cells. (c) The viruses may also use TNTs with or without vesicles to transfer their components (nucleic acid, structural proteins) to neighboring cells during the later stages of infection. TNTs allow the viral structural components to avoid contact with the extracellular matrix (ECM) which can expose them to immune cells, proteases, drugs, or nearby active phagocytic cells. **(B)** Significance of TNTs bridges during virus-host interactions. (a) Actin-rich filopodia aid in viral surfing to reach the target cell. (b) TNT bridges mediate a long-distance transfer or movement of the virions from the initial infected sites to broaden the virus tropism. (c) Viruses can also use cellular cargos (endocytic vesicle, signaling molecules) to transfer their components or intact virions to reach neighboring cells safely. (d) TNTs also provide essential means to aid the process of viral spread to nearby adjacent cells.

Many medically important viruses, such as the influenza virus, human immunodeficiency virus (HIV) and herpes simplex virus, can evade host immunity and avoid pharmaceutical targeting by using TNTs to pass their genomes to naive cells (12, 52). In addition to protecting the viral components from the extracellular environment, utilizing TNTs to propagate infection is more energetically favorable than conventional methods. The production of viral components, such as a viral genome and the various proteins involved in budding, host cell binding, and fusion can be avoided by inserting viral genomes directly into the cytoplasm of a host cell (**Figure 4A**). Additionally, TNT transport is significantly faster than extracellular transport. Mitochondria that contain infectious materials have been recorded traveling 7 nm/sec (37). The speed of transport combined with rapid rate of TNT polymerization (0.2 μm/second), makes TNT travel vastly more efficient (20, 53). TNTs are also incredibly large, having diameters upward of 200 nm, and can accommodate large macromolecules and cellular organelles (3). In addition to transporting viral components (**Table 1**), they can also be used as a track for virions to surf on, reducing the time it takes to spread from cell to cell (17, 59). For example, the formation of TNTs between epithelial cells and fibroblasts is likely to disseminate herpesviruses over long distances (54). Such transfer may be preferential over the traditional receptor requirements and broaden the host cell tropism.

**Table 1:** List of the viruses and the viral components including the cell-type associated with TNT bridges are listed.

Viral components on TNT bridges	Virus	Cell-type	References
gE (*glycoprotein)*, VP26 (*capsid protein)*, and US3 (*tegument protein)*	BoHV-1	Primary fibroblasts, KOP cells	(54)
p24 (*capsid protein)*	HIV	Macrophages	(12)
Nef (*accessory protein)*	HIV	B lymphocytes	(38)
gag (*structural protein)*	HIV	MDMs	(22)
P8 (*accessory protein)*	HTLV	Jurkat T cells	(55)
P (*phosphoprotein)*	HMPV	BEAS-2B	(56)
Extracellular virions	MLV	Cox-1, XC, HEK	(17)
RNP and polymerase	Influenza A	MDCK, A549	(57)
RNA	Influenza A	MDCK, A549	(52)
Whole virions	PRV	Swine testicle cells	(58)

**Figure 5:**
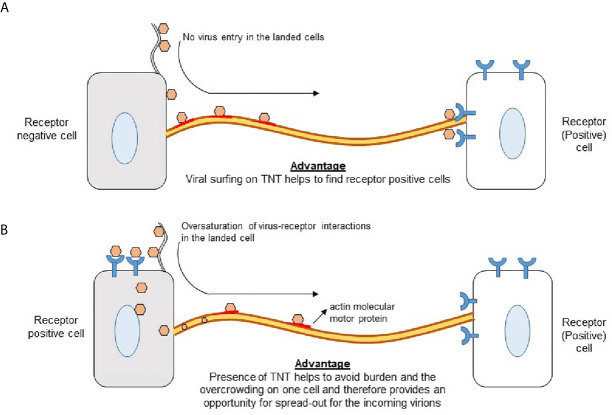
Significance of TNTs to promote virus-cell interactions **(A)** Virus landing on receptor negative cells induces TNT bridges which aids in viral surfing to find the receptor positive cell for cell entry. **(B)** Virus landing on receptor positive cell results engagements of virus-cell interactions *via* receptor leaving an opportunity for the coming virions to use TNTs to find the receptor positive cells for cell entry.

TNTs serve as a convenient network for viral exploitation because they offer a protected, direct highway from an infected cell to naïve cells (**Figure 4B**). Intracellular viral spread *via* TNTs provides critical protection from circulating immune cells and avoids virus-cell interactions that may alert host defenses. Furthermore, TNTs allow for infectious particles that initially attach to non-permissive cells to spread to vulnerable cells and promote infection (**Figure 5**). Understanding the mechanisms of TNT utilization for effective viral infection is vital for better treatment of human pathogens. An understanding of TNTs may also elucidate mechanisms by which viruses are able to remain dormant inside a host, effectively avoiding immune and pharmaceutical targeting.

### Herpesviruses

Herpesviruses cause many diseases in humans from blistering diseases to encephalopathy and blindness. Accordingly, herpesviruses have always been the focus of intense research to form antiviral drugs. Unfortunately, drugs like acyclovir are useful only after infections have begun (60). These antivirals prevent further viral replication to decrease the number of outbreaks but cannot cure the disease and allow for the virus to remain dormant in the trigeminal ganglia. One mechanism that may make treating this affliction more difficult is the viral utilization of TNT networks. Using fluorescently labeled viral proteins and time-lapse confocal microscopy, a recent study demonstrated that herpesviruses receive protective benefits *via* travel by TNTs (54). Despite the presence of neutralizing antibodies, alpha-herpesvirus BoHV-1 (bovine herpesvirus 1) is capable of transmitting viral particles and cellular organelles along TNTs produced by bovine primary fibroblasts and oropharynx cells (KOP) (54). The viral kinase US3 of pseudorabies virus (PRV) was sufficient to form a very stable TNT which lasted up to 24 hours (58, 61). Furthermore, the infected cells were shown to spread the virus *via* TNTs to uninfected cells. Although US3 protein is conserved among alpha herpesviruses (subfamily of herpesviruses), other members of the herpesvirus family, such as gamma herpesviruses exemplified by murine gamma herpesvirus 68 (MHV-68) and Epstein-Barr virus (EBV), have also been shown to induce and exploit TNT for viral spread using gp48 and ORF58 (62–64). In the above examples, it is believed that herpesvirus proteins affect Rho GTPase signaling to promote the TNT formation (61, 65, 66). This evidence suggests that viral utilization of TNTs must be targeted to best combat these herpesviruses.

### Influenza A

Influenza is a major cause of mortality even in the developed world (67). Additionally, different strains of the virus emerge each year, some virulent enough to cause pandemics (67). The members of orthomyxoviruses (Influenza A virus and the parainfluenza virus) as well as members of paramyxovirus (measles) families have been shown to induce TNTs to aid in intracellular viral spread and syncytia formation. The virus spread was very meaningful since it happened even in presence of virus neutralizing antibodies or in the presence of a neuraminidase inhibitor (52). Infected cells transferred viral proteins and genomes to uninfected cells *via* TNTs. This method allowed the virus to evade the inhibitory effects of the antibody and Oseltamivir therapies. However, treatment with Cytochalasin D, an actin polymerization inhibitor, successfully reduced the spread of viral proteins and thus alleviated the infection (52). These results underline the role of TNTs in immune evasion and viral spread during influenza virus infections.

### Human Immunodeficiency Virus

Human macrophages were first shown to increase TNT production upon HIV infection, and TNT production was correlated with viral replication (12). HIV particles also have been found inside TNTs during infection (12). Interestingly, both extracellular surfing and intracellular endocytic transfer of the virions were observed. These findings suggest that HIV has evolved a mechanism to upregulate TNT to facilitate the spread of infection. The virus hijacks TNTs to shuttle virions between cells. While HIV can induce formation of TNT in macrophages, HIV-infected T cells do not increase TNT production (41). Nonetheless, the virus still uses TNTs to travel between and infect T cells (41).

The mechanism by which HIV induces TNT formation in blood monocyte-derived macrophages requires the HIV accessory protein Nef and the cellular protein M-Sec (22). Nef interacts with the Rho GTPase RalA and the exocyst complex during this process. Nef-deficient HIV could not induce macrophages to produce TNTs (22). This signaling pathway also contains M-sec as it was impeded in a cloned cell line lacking M-sec. By using a small molecule which inhibits M-Sec mediated TNT production, researchers were able to reduce HIV infection by half (22). This reduction was not seen in Nef-deficient mutant HIV-1 or in a CD4^+^ T cell line which is deficient in M-Sec (22). These results support the existence of a Nef-M-sec signaling pathway that is vital for TNT formation and subsequent viral release. In addition, it has also been demonstrated that Nef association with the Rac1/Cdc42 effector p21-activated kinase 2 (PAK2) along with the exocyst complex leads to upregulation of MyoX, which is required for TNT induction (68, 69). Therefore, it has been suggested that Nef protein is able to hijack the cell’s sensing and intercellular-communication machinery by increasing MyoX-dependent TNT formation (69). Although, the overall cause of the trigger during Nef mediated TNT formation is not clear, mass spectrometry analysis of Nef immunocomplexes from Jurkat cells have recognized exocyst complex proteins as a critical effector of Nef-mediated enhancement of TNT formation (69). Interestingly, a recent study also showed that HIV and *Mycobacterium tuberculosis* in a co-infection model trigger the TNT formation *via* IL-10/STAT-3 signaling (11). Tuberculosis-associated microenvironments during an HIV infection induce the upregulation of the Siglec-1 receptor protein which associates with thick TNTs (70). TNTs containing Siglec-1 were more likely to carry HIV proteins and more stable than thinner TNTs (70).

Interestingly, HIV-induced TNTs have been found to contain connexin-43, a key protein in the formation of gap junctions, at their synaptic contacts (71). Connexin-43 has been independently reported to support TNT formation (72). Dyes micro-injected in HIV-infected macrophages were able to be transported through TNTs and their terminal gap junctions to uninfected macrophages (71). As the dye transfer was inhibited using gap junctional blockers, gap junctions formed during TNT coupling of cells can facilitate the movement of intracellular components (71). Gap junctions at the ends of TNTs and the TNTs themselves were shown to be essential for HIV transmission between human macrophages. Gap junctions on TNTs may be used to communicate depolarization signals as well as one study reported that only connexin-43^+^ TNTs were able to participate in electrical coupling (15). Thus, gap junctional TNTs may play a role in neuronal signaling as well.

### Human T Cell Leukemia Virus Type 1 (HTLV-1) -1

HTLV-1 is an oncogenic virus that can cause T cell lymphomas and tropical spastic paraparesis (73). HTLV-1 spread is particularly dependent on cell-to-cell contact as extracellular particles have low infectivity (74). Accordingly, HTLV-1 may benefit greatly from TNTs. Methods by which the virus can spread from cell to cell include lipid rafts, viral biofilms, and other cellular conduits such as TNTs (55). The HTLV-1 p8 protein has been shown to down-regulate TCR activity (75). It also increases TNT connections between T cells and itself is transported *via* these connections (76). The p8 protein also increases T cell clustering by increasing the expression of lymphocyte function-associated antigen-1 (LFA-1) on the cell surface (76). The p8-mediated TNT formation was shown to be independent of the Tax protein which can polarize microtubule-organizing centers in the cell. Interestingly, role of the p8 protein in HTLV-1 has been compared to Nef in HIV-1 with regards to localizing to the immunological synapse and impacting TCR functionality (76). Overall, p8 induces T cell anergy and stimulates TNT formation amongst T cells to promote viral spread and antagonize immune recognition processes.

### Human Metapneumovirus (HMPV)

HMPV is a member of the paramyxovirus family and can cause upper and lower respiratory infections by infecting cells of the respiratory tract, such as bronchial cells (77). Like other viruses previously discussed, HMPV can use TNTs to propagate infection. HMPV viral spread was shown to be dependent upon actin, not microtubule, polymerization (56). The HMPV P protein co-localizes with actin and mediates the production of TNT structures (56). Treatment with neutralizing antibodies or a lack of heparan sulfate on target cells inhibits entry of HMPV (78). However, the virus was still able to spread to uninfected cells effectively *via* actin-rich projections consistent with TNTs. Inhibition of Rho GTPases Cdc42 or Rac1 decreased TNT formation and successfully reduced HMPV spread. Thus, HMPV exhibits a similar reliance on TNTs as influenza. Overall, these findings further support the up-regulation and exploitation of TNTs by many viruses to spread and infect target cells.

### Severe Acute Respiratory Syndrome Coronavirus 2 (SARS-CoV-2)

**Figure 6:**
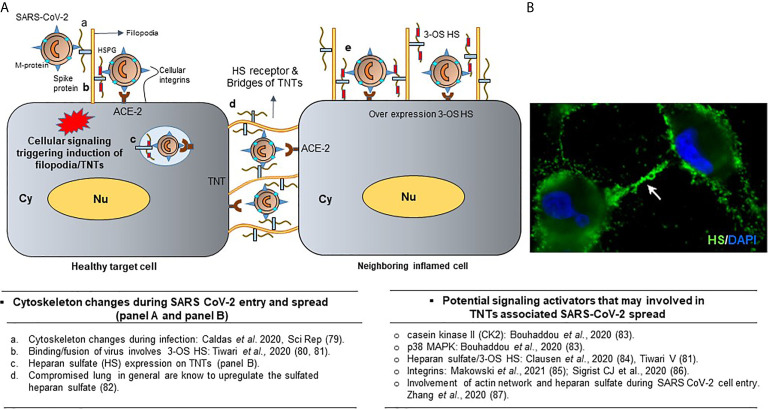
Significance of TNTs during SARS-CoV-2 infections. **(A)** Cellular signaling leads to the heparan sulfate (HS) rich projections in the form of filopodia which facilitates viral entry. Presence of viruses on TNTs bridges during spread. Overexpression of heparan sulfate and chemokine in the inflamed cells are shown. **(B)** Surface expression of HS receptor (green) on the TNT bridges using anti-HS antibodies (US biological) along with the DAPI stained nucleus is shown. Finally, the literature associated with the cell signaling during the activation of cellular protrusions during SARS CoV-2 infections are highlighted.

SARS-CoV-2 induced surface disruption at the level of the host cell’s actin cytoskeleton legitimizes the potential that other surface perturbations, such as the eruption of F-actin containing TNT bridges at the cell surface, may assist SARS-CoV-2 widespread dissemination. In fact, previous studies have shown that the cytoskeleton network plays an important role during the entry, replication, and maturation process of coronaviruses, including SARS-CoV-2 (**Figure 6**) (79, 88, 89). A recent study by Caldas et al., 2020 provided the first evidence of the SARS-CoV-2 mediated viral surfing on filopodium and the occurrence of a thin (< 0.7 μm) strand of F-actin containing tunneling nanotube (TNT) using high resolution electron microscopy (79). Viral particles adhered to cell surface protrusions that were shown to connect two cells. This observation suggests viral “cell surfing” previously described by other enveloped viruses such as HIV and human metapneumovirus. This mechanism is presumed to allow the *in vivo* penetration of viruses in mucosal surfaces that display microvilli-rich cells. SARS-CoV-2 infected cells have also recently been found to have strong upregulation of casein kinase II (CK2), a protein kinase that induces phosphorylation of cytoskeleton protein targets like α-catenin and motor protein myosin IIa (83). In this study, Bouhaddou et al., 2020 demonstrates that SARS-CoV-2 infected cells produced longer actin-rich, CK2-containing filopodia than those filopodia found on noninfected cells, and that these infected cell filopodia possessed viral particles within themselves (83). Possible TNT activity has also been found in the topographic changes to the surface of cells infected with related beta-coronavirus SARS-CoV, namely in the collection of progeny virus particles prepared by Golgi sacs for export to the external surface of the cell (88). Viral progeny, while frequently disseminated from hijacked cells *via* exocytosis, may also collect in these localized regions for transport *via* actin-rich bridges, like TNTs, across cells. Similarly, other single-stranded RNA viruses have demonstrated TNT developmental behaviors, including the gastroenteritis virus (TGEV) which has been found to induce F-actin polymerization, or protrusive growths from the cell surface, and SARS-CoV and murine hepatitis virus, which induce cell membrane interruptions and filopodia formation like the CK2 protrusions off SARS-CoV-2 infected cells (89). This evidence suggests that SARS-CoV-2, like many of the viruses to which it is related, uses TNTs to propagate its pathogenesis, which may offer some insights into its ability to affect multiple organs and cause a diverse array of complications throughout the body. The benefit of understanding TNT formation by SARS-CoV-2 may also have further clinical significance. For instance, when analyzing the treatment of a systemic inflammatory disease known as acute respiratory distress syndrome (ARDS) and sepsis with mesenchymal stem cells (MSCs), TNTs have been found to enhance MSC mitochondrial transfer, improving macrophage oxidative phosphorylation and phagocytosis and strengthening the body’s immune response to invading pathogens within cells (90). Because SARS-CoV-2 has been associated with similar inflammatory syndromes, better referred to as multisystem inflammatory syndrome in adults and children (MIS-A and MIS-C, respectively), identifying viral TNT development in infected cells offers a target for mitigating viral spread between cells and slowing disease advancement (91). Furthermore, it could also offer a new and highly-cell specific means of distributing therapeutic treatments through the nanotubules themselves. Taken together, understanding hijacking of a host cell by SARS-CoV-2 is a critical step toward developing effective therapeutics and prophylactics.

**Figure 7:**
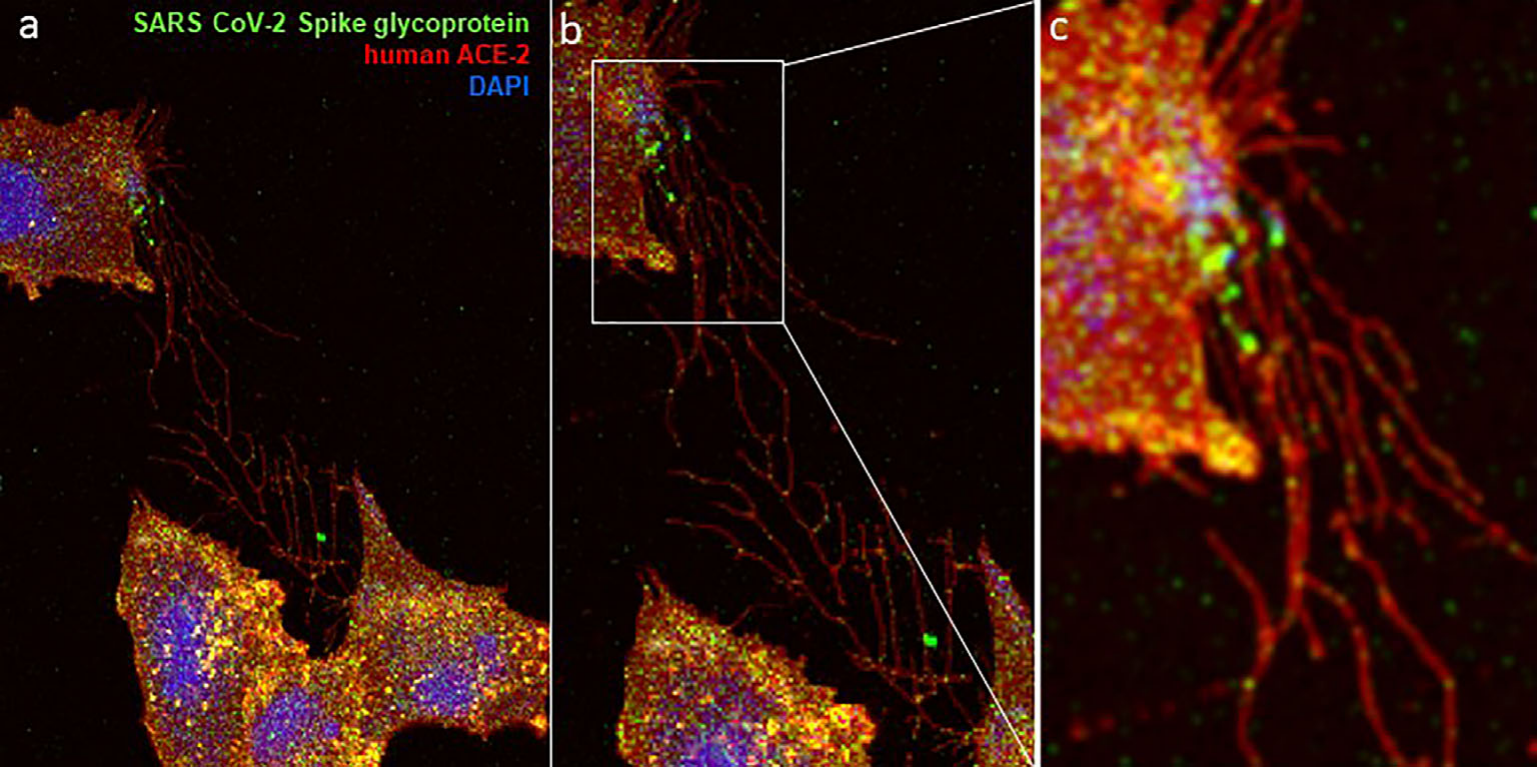
Visual evidence of TNTs network is shown during the co-culture of a target cells (expressing the human ACE-2/heparan sulfate receptor) and the effector cell (expressing the SARS CoV-2 spike glycoprotein). Co-culture of heparan sulfate rich Chinese hamster ovary (CHO-K1) cells expressing GFP-spike glycoprotein with the CHO-K1 cells expressing RFP-tagged ACE-2 cells show the complex network of TNTs between the cells **(A–C)**. A higher GFP signal for SARS CoV-2 spike glycoprotein at the cellular tip including within the TNT bridges is highlighted in panel b and panel c. Confocal microscopy on the fixed co-cultured CHO-K1 cells was performed after 48 hours post-mixing using a 60× oil objective (Nikon Eclipse Ti).

This entry pathway of SARS-CoV-2 is facilitated by the cell surface heparan sulfate proteoglycan (HSPG), which together with human ACE-2 receptors enhance viral attachment and cell entry (**Figure 6**) (80, 84, 92). A recent study by Zhang et al., 2020 found that a collection of FDA-approved drugs was effective in preventing SARS-CoV-2 cell entry at distinct steps (81). These drugs included Mitoxantrone, Raloxifene, and Piceatannol, which are known to bind to heparan sulfate. Similarly, drugs such as Sunitinib, BNTX, and Tilorone target the actin cytoskeleton and lysosomes (81). Since these drugs target heparan sulfate and actin cytoskeleton receptors during virus entry and trafficking, it is logical that they may inhibit virus transfer *via* TNTs. In fact, the co-culture of cells expressing spike glycoproteins of SARS-CoV-2, along with the cells expressing human ACE-2 and heparan sulfate especially at the tips, resulted in induction of the widely distributed TNTs between cells (**Figure 7**). The presence of TNTs during SARS-CoV-2 infection is supported by an upregulation of casein kinase II (CK2) (89) and heparan sulfate (87), a key enhancer of actin cytoskeleton and the cellular receptor for virus entry may have far reaching clinical outcomes with profound and widespread dissemination of SARS-CoV-2 *via* TNTs affecting multiple organs and the disease severity as consistently observed with COVID-19 patients (82). Clearly, understanding the hijacking of host cell TNTs by SARS-CoV-2 during an early or late stage of infection seems worthy of future investigation in order to develop effective measures towards therapeutics and prophylactics.

### Mitochondrial Transfer During Infection

Mitochondria are important regulators of apoptosis and cell survival. In a study by Wang and Gerdes, UV light-damaged pheochromocytoma cells co-cultured with undamaged cells avoided apoptosis (93). The damaged cells received mitochondria through TNTs, and although they released cytochrome c from the damaged mitochondria, they did not activate the caspase-3 apoptosis pathway (93). To further demonstrate TNTs role in this process, cells with defective nanotube formation could not inhibit apoptosis (94). The transfer of mitochondria from healthy cells to damaged cells *via* TNTs is beneficial for viruses. Mitochondrial transfer promotes viral survival, factory production, infection rate, and redistribution of mitochondria; overall, leading to an increase in infectious viral particles. For example, porcine reproductive and respiratory syndrome virus (PRRSV) can not only induce TNT formation in infected cells but also spread to uninfected cells *via* proteins that associate with mitochondria (94). The same process that rescues damaged cells ultimately spreads more virus.

## Role of TNTs in Cancer

TNTs play an important role in the progression of many well-studied types of cancer (4). For example, in the B-cell cancer acute lymphoblastic leukemia (ALL), leukemia cells transport signaling molecules through TNTs to communicate with mesenchymal stromal cells and influence the molecules that they release, effectively modifying their environment (95, 96). The mesenchymal stromal cells secrete the pro-survival signals monocyte chemotactic protein 1, CXCL10, and interleukin 8 to the cancer cells (96). Interestingly, disrupting TNT formation by targeting actin polymerization disrupts pathogenesis and sensitizes the cells to prednisolone (96). The ability of cancer cells to communicate with regulatory cells and cytokine-producing cells gives the cancer more power to control its microenvironment. The cells can upregulate the release of pro-growth cytokines and induce a more malignant phenotype. It is paramount that future pharmaceuticals consider this component of cancer pathogenesis for new drug development. With cancer causing a significant global health burden, investigations regarding the role of these intimate cytoplasmic bridges have become a promising area for targeted anti-cancer therapies.

*In vitro* studies utilizing human cancer cells have provided evidence of various mechanisms through which TNTs modulate proliferative and invasive potential of cancers. TNTs facilitate homocellular communication between malignant and normal mesothelial cells (97). TNTs can also mediate the bidirectional exchange of cytosolic membrane components, proteins, and mitochondria (97). Additional co-culture studies have further explored the impact of TNT-mediated organelle transfer. Mitochondria have reported to be transferred through nanotubular connections (100-200 nm wide) between RT4 (less invasive) and T24 (highly invasive) bladder cancer cells (98). The researchers linked mitochondrial trafficking resulting in activation of mTOR and downstream signaling pathways to increased invasive potential of RT4 cells (98).

Since the current model of cancer physiology concedes that tumor growth is influenced by complex interactions between cancer cells and various stromal cell types (i.e. mesenchymal stem cells, endothelial cells, fibroblasts, macrophages), cell-to-cell communication structures have become a target of investigation in response to acquired drug resistance (99). Utilizing ovarian cancer cell lines (SKOV3 and OVCAR3) and breast cancer cell lines (MDA-MB231 and MCF7), Pasquier et al., 2013 identified crosstalk between tumor cells and stromal cells, with nanotubular bridges functioning as a conduit for cell-to-cell communication. Heterocellular mitochondrial transfer facilitated by TNTs was associated with increased chemoresistance in cancer cells (99). The researchers postulated that mitochondrial trafficking may promote resistance *via* various mechanisms including prevention of apoptosis, reduced generation of reactive oxygen species (ROS), and increased rescue of cells with dysfunctional mitochondria (99). Using *in vitro* pancreatic and ovarian cancer systems, Desir et al. discovered efflux of doxorubicin (a topoisomerase II inhibitor) mediated by TNTs, with drug-dependent increase in TNT formation (100). Furthermore, the researchers indicated that nanotubular bridges enabled drug resistance through mechanisms of drug redistribution, in addition to previously studied means of mitochondrial and O-glycoprotein transfer (100).

In addition to facilitating organelle transfer, studies have implicated nanotubular structures in potentiating angiogenesis and creating sustainable environments for tumor cell proliferation and metastasis. TNTs mediate pericyte vascularization and collateralization in fetal cerebral cortex and human glioblastoma samples (101). The researchers indicated that nanotubular bridges modulate physiologic brain angiogenesis; however, they are also involved in pathologic vascularization that promotes tumor micro-environments in the brain (101).

Taken together, TNTs present a novel mechanism of cell-to-cell communication in cancer. Further research clarifying the mechanisms of chemoresistance through mitochondrial transfer or macromolecular exchanges are necessary in developing targeted anti-cancer therapies. Additionally, supportive *in vivo* investigations are critical in adequately understanding the complex interplay of nanotubular connections in early pathogenesis and spread of human cancers.

## TNT in Chemotherapeutic Resistance

It has been shown that cancer cells may develop resistance to chemotherapeutics even without prior exposure to them. One explanation for this phenomenon is that TNTs mediate communications between cancer cells exposed and not exposed to chemotherapeutics which selects for progeny that are resistant. This mechanism of resistance has been demonstrated in pancreatic cancer cells that were harvested from patients and then treated with the popular chemotherapeutic drug doxorubicin (100). Doxorubicin induces TNT formation in a dose-dependent fashion as measured by multiphoton fluorescence microscopy. Chemo-sensitive cancer cells could transport doxorubicin to resistant cells *via* TNTs and vice versa (100). These results add chemotherapeutics to the long list of cellular stresses able to induce TNT formation but also highlight another method by which cancer cells utilize TNTs. The transfer of drug not only reduces the concentration that the target cells are receiving but also exposes neighboring cells to the effects of doxorubicin at lower dosages. For sensitive cancer cells, TNTs effectively increase the likelihood of survival by lowering the dosage present in the cellular microenvironment (100). For resistant cells, the transfer of doxorubicin kills sensitive cells, reducing the number of cells competing for nutrients with the resistant subpopulation and increasing the chances of resistant cells reproducing in the next generation (100). While doxorubicin is just one example, this phenomenon likely applies to many pharmaceuticals and should be part of future considerations for cancer treatment.

Glioblastomas provide more evidence of the role of TNT-like structures in drug resistance. Glioblastomas are particularly nasty cancers with a median survival rate of 1 year post diagnosis and a 5-year survival reported at 5-10% in high grade cases (102). When untreated cells are co-cultured with cells that are treated with temozolomide and radiation therapy, the unexposed cells demonstrated decreased rates of apoptosis upon treatment (103). This effect is thought to be mediated through connexin-like connections such as those of TNT. Because of the similarities between the cell-cell connections made by connexins and TNTs, it is possible that protection conferred by connexin junctions could also be mediated, over greater distances, with TNTs. For example, the gap junction-mediated transport of Cx43 between dopaminergic neuroblastoma cells has been shown to decrease the efficacy of the parkinsonian toxin 1-methyl-4-phenylpyridine (MPP^+^) (104). Thus, the transport of numerous cell products or drugs through TNTs may reduce or enhance the effectiveness of certain therapies.

It may be possible to exploit TNT-mediated transport of molecules between cancer cells. Tumor cells expressing HSV thymidine kinase (TK) can be killed with ganciclovir treatment (105). However, methods using a transgene to express TK in tumor cells suffer from low efficiency (105). Researchers have found that ganciclovir could pass from TK-expressing cells to cells lacking TK expression, improving the efficacy of the therapy (105). Ganciclovir transport was reported to be mediated by gap junctional intercellular communication (80). Given the similarities between gap junctional transport and TNTs, we speculate that TNTs could also play a role in transferring ganciclovir or other toxic compounds between cancer cells which would provide a unique opportunity for targeted drug delivery.

## TNT Role in Neurodegenerative Diseases

The significance of TNTs extends beyond viral infections and cancer as they play a role in the pathogenesis of numerous neurodegenerative diseases, many neurodegenerative diseases are the result of an accumulation of misfolded proteins. There exists evidence that disease such as Parkinson’s, Huntington’s, and Alzheimer’s diseases can be propagated through passage of defective proteins through TNTs. In a model of Parkinson’s disease., α-synuclein fibrils were transported through TNTs inside of lysosomes from defective cells to healthy cells, contributing to α-synuclein aggregation in the latter (106). Misfolded tau proteins, which are involved in the pathogenesis of Alzheimer’s disease, can travel inside of TNTs (107). Furthermore, cells which receive the tau proteins exhibit increased TNT formation. In both Parkinson’s and Alzheimer’s studies, TNTs propagate the disease by spreading α-synuclein fibrils and tau proteins respectively to healthy cells, like viral particle spread. A summary of the uses of TNTs in neurodegenerative disease and cancer is provided in **Table 2**.

**Table 2:** Significance of TNTs in cancers and neurodegenerative diseases including the cell-type associated with TNT bridges are listed.

Uses of TNT Bridges	Cell-type	Significance	Reference
Transfer of Mitochondria	Pheochromocytoma (PC) 12	Survival mechanism by stressed cell	(93)
Transfer of vesicles and proteins	Human primary tumors/cancer	Cancer cell pathogenesis and invasion	(97)
Transfer of Tau protein	Rat primary embryonic cortical neurons	Alzheimer’s disease	(108)
Fibrillar α-synuclein	CAD cells	Parkinson’s disease	(106)
Rab8a/Rab11a	Schwann cells	Peripheral nerve regeneration	(106)
Hypoxia-induced TNT formation	Chemo resistant ovarian cancer cells	Malignant and tumor cell interactions	(109)
Signaling	Acute lymphoblastic leukemia (ALL) cells	Communication mechanism	(96)
Transfer of H-Ras, a small GTPase	B and T cells	Increase in p-ERK1/2 levels in the acquiring T cells	(110)
Fas signaling	CD4+ T cells	Intracellular communication and death signaling	(111)
Calcium signaling	Human retinal pigment epithelial (RPE) cells	Electrical coupling	(112)

The pathogenesis of Huntington’s disease pathogenesis involves expanded CAG repeats in the huntingtin gene (113). The translated huntingtin protein contains a longer polyglutamine stretch which results in misfolding and aggregation (113). Huntingtin aggregates inhibit numerous cell processes (113). Murine neuronal cells expressing the mutant huntingtin gene spread the aggregates to co-cultured cells. This process required cell-cell contact, but the proteins were not spread *via* the culture medium. Instead, the huntingtin aggregates were transported through TNTs. Furthermore, mutant huntingtin stimulated TNT formation in a parallel mechanism to those in Parkinson’s and Alzheimer’s diseases (114).

## TNTs - *In Vivo* Significance and Challenges

There are growing evidence for the involvement of TNTs in multiple physiological and pathological disease processes *in vivo*. For example, neural crest migration which is a developmental event critical for maintaining the facial skeleton homeostasis including nervous system development, proceeds *via* TNT formation. In a recent study the *in vivo* labeling of chick premigratory neural crest cells demonstrated the significance of lamellipodia as well as short, thin filopodia (1-2 μm wide) for initiating the local contacts between the migrating cells (<20 μm) (115). Non-local, long distance contact (up to 100 μm) was initiated by filopodia that extended and retracted, extended and tracked, or tethered two non-neighboring cells for directional guidance. Similarly, TNT involvement during the differentiation of multi nucleated giant cells such as placental trophoblast, myotubes, and osteoclasts has also been suggested to be critical for maintaining the local tissue homeostasis (115–117).

In the field of cancer biology, the tumor cells acquiring an enhanced metabolic plasticity, migratory phenotypes, angiogenic ability, and therapy resistance -all mediated *via* TNTs- contribute directly to enhanced aggressiveness of various forms of cancers (5). Supporting this further, extensive formation of TNTs has been reported in several cancer *in vitro*, *ex vivo*, and *in vivo* models.

Quite interestingly, a study by Jackson et al. (9) has shown antimicrobial effects of mesenchymal stromal cells (MSC) by transferring their mitochondria to macrophages using TNTs. In this study, using a mouse model of *E. coli*‐induced pneumonia it was demonstrated that mitochondrial transfer *via* TNTs improves macrophage mitochondrial function and ATP turnover *in vitro* and enhances macrophage phagocytic capacity both *in vitro* and *in vivo* highlighting an important mechanism of the antimicrobial effect of MSC *in vivo*. Similarly, putative TNT formation between the dendritic cells and bone marrow-derived MHC class II (positive) cells in the corneal stroma has also been observed (10, 42, 43). Interestingly, the number of TNTs was significantly increased in corneas subjected to trauma and LPS, which suggests that nanotubes may have an important role to play in an immune privileged site. The later studies are interesting and may shed light on TNT’s unique role in ocular infections such as herpetic stromal keratitis. Another interesting physiological process where TNT-dependent electrical signaling could attribute its significance is during the wound healing process. Membrane depolarization at the leading edge of wounds has been shown to connect adjacent cells *via* TNTs activation of PI3 kinase signaling suggesting a supportive role of TNTs in wound healing, which involves F-actin remodeling (118). Similarly, TNT formation and induction have also been observed following injury, trauma or chronic tissue stresses (38).

Clearly, it has been very challenging to study TNTs *in vivo*. Absence of well-defined *in vivo* molecular markers to specifically identify TNTs and their associated mechanisms involving precise transfer of key molecules of interest either during homeostasis or disease pathologies has made discoveries difficult. Complicating this further, studying the three-dimensional nature of TNTs under the complex and relatively unstable extracellular tissue environment requires high resolution tissue imaging techniques and unique collaborative expertise involving clinicians and scientists to understand the precise link between the disease and the TNTs. Taken together, understanding the molecular triggers for TNT genesis *in vivo* and the associated molecular information passed between cells *via* TNTs *in vivo* constitute an important but challenging area for future biomedical research. Our ability to manipulate TNTs *in vivo* under both normal physiological conditions as well as pathological scenarios will be helpful to understand the cellular communications in tissue environment and eventually be used for improving cellular functions to achieve better human health.

## TNT Targeting Therapies

TNT involvement in numerous pathologies makes it a prime target to treat diseases ranging from cancer to dementia. However, because the TNTs are primarily composed of actin, which is essential for various cellular processes and maintenance of the cytoskeleton, a delicate balance must be preserved when targeting TNTs. Therefore, the therapies must be refined to impact only pathological processes while preserving normal function. Studies have found that the mTOR inhibitor everolimus or diabetes medication metformin inhibit TNT formation *in vitro* (109). Everolimus is currently FDA-approved to treat multiple forms of cancer: breast, pancreatic, lung, gastrointestinal, among others (119). Cytochalasin B and D inhibit actin polymerization which prevents TNT formation (9). However, as general inhibitors, they lack the specificity required for effective therapeutic use. The nucleoside analog cytarabine was also reported to inhibit TNT formation and is currently in use to treat variants of leukemia (120, 121).

Many chemotherapeutics inhibit TNTs as part of their mechanism of action, and novel TNT inhibitors could have the potential to be anti-cancer therapies as well.

## Conclusion/Discussion

The understanding of TNT regulation and function in both physiological and pathological states is of utmost importance in treating many different diseases. The evolution of cells to create TNTs to maximize cellular communications either during homeostasis and or during stress response to improve the cell survival by cargo transport to transferring energy, Ca2+ ions, mRNA and cellular organelles while in parallel evolution of pathogens to adapt and exploit TNTs are equally fascinating outcomes highlighting the TNTs potential which needs to be fully harnessed as a therapeutic target. Further, multiple studies have provided *in vivo* evidence to support the role of TNTs in pathophysiology and several forms of the disease including their presence between immune cells either in the lymph nodes (20), and or between the dendritic cells in the mouse cornea (43). Data generated from various cancer model studies have also provide compelling evidence regarding the contribution of TNTs (5) or capable of crossing the dense tubular basement membrane in the kidney of the cystinosis mouse model (44) or in their cornea and thyroid (43, 122). One major issue in performing these *in vivo* and *ex vivo* studies is the difficulties in identifying the precise nature of the structures and clearly determining their role in the transfer. Nonetheless, data generated from various laboratories reported evidence of TNT-like structures in brain tumors, and in *ex vivo* hematopoietic stem cells, lung, and ovarian cancers. Also, TNT-like structures were found in human macrophages present in lymph nodes obtained from HIV-infected individuals with HIV reactivation.

The similarities and the differences in the virus transfer *via* TNTs are also worth noting. As per the current hypothesis during an early phase of viral entry many medically important viruses from different families exploit the bridges of TNTs as a super spreader to move rapidly across long distances (54). It is widely accepted that many viral infections trigger the activation of highly conserved signaling Rho GTPases dependent Cdc42/Rac-1 pathway (39). The later event aid in the actin polymerization containing F-actin and the network of microtubules which in turn favors the virus transfer. The absence of microtubules on TNTs supporting viral spread is also documented (39). Interestingly, TNTs also offer a unique viral surfing platform for viruses to move even on outer extracellular surfaces which could help their movement a lot quicker than the densely rich intracellular environment (12). This mechanism has been shown in HIV and herpesviruses, but it is quite possible to have similar mechanism exist with other viruses as well. In this process it is quite possible that the ubiquitously expressed heparan sulfate (HS) receptor on TNTs together with the associated fibroblast growth factor (FGF), and integrin signaling may be involved to aid in the viral spread. Although the precise mechanism with the direct receptor involvement on TNTs to promote viral spread remains unknown. In fact, many viral structural proteins have ability to trigger the induction of TNTs (39), but their formation depends on the interaction of the virus receptor or the viral ligand depending on the infected or the uninfected cell. This could be varied depending on the type of viruses including the target cell. In contrast, the role of non-structural protein to trigger TNTs is also documented which clearly suggest that the involvement multiple other co-factors and possibly ubiquitous HS related signaling event may favor this process. However, it is worth investigating if the virus-induced TNTs could display either a unique marker or an increased expression of pre-existing receptor (HS and other adhesion molecules). Given the fact that many viruses move intracellularly *via* TNTs it is clear that they all acquire one crucial benefit by being protected against extracellular circulating immune cells, and or pre-existing antibodies or even viral entry blockers. Interestingly TNTs also play a vital role during the viral genome replication or during viral egress. This is evident from the fact that TNTs enables the transfer of the entire virions (example HIV, herpesviruses etc.), individual viral proteins (HIV; p24; herpes envelope glycoprotein E (gE), capsid protein VP26, and tegument protein Us3), as well as the viral genomes (example, influenza virus) to the recipient cells (39, 52). Therefore, the transfer of complete virus particle and or viral cargos suggest that viruses have evolved a unique mechanism and strategy to spread at multiple stages of the virus life cycle. Nevertheless, it is clear that virus ability to infect and replicate successfully inside host dependent on multiple other factors and TNTs are one of them. One additional interesting question that remains a mystery are the specialized elements of communication involved to benefit the virus *via* TNTs. In this regard shedding additional light on highly conserved virus-host cell receptor interactions across multiple viruses, identifying exclusive TNTs marker, and developing high resolution *in vivo* imaging tool will help to understand the role of TNTs in viral infection much better.

Just as viruses and other pathogens have evolved to develop the capability to exploit TNTs, so too must modern medicine develop therapeutics that target the production of TNT and the spread of pathogens inside of and atop TNTs. While we currently have the capabilities to inhibit actin polymerization, we must next develop less toxic and more specific inhibitors of TNTs while maintaining normal cellular function. Such therapies would have implications not only in the realm of infectious disease, but also in cancer therapy and in the treatment of neurological disorders. Exploring the signaling, production, locations, and properties of TNTs may also prove to be valuable in understanding the timeline of disease pathogenesis. Examples include a cancer developing resistance to certain therapies or a virus switching methods to spread to naïve cells. Additionally, the ability of certain cells to produce TNTs with specific traits and function may also influence tissue tropism for viruses and other disease-causing agents. Medicine must adapt as we make advances in understanding the complex mechanisms that underlie cellular physiology and pathophysiology. In this way, we can create more comprehensive therapies and produce better outcomes for patients.

## Author Contributions

VT, RK, GR, and AS wrote the manuscript. DS and VT edited the manuscript. Figure was developed by VT. GR wrote the SARS-CoV-2 section and assisted with the new figures associated with SARS-CoV-2 including references. All authors contributed to the article and approved the submitted version.

## Funding

This work was supported by the Midwestern University Research Start-up Funds to VT. The Support by NIH grants (R01EY029426 and P30EY001792) to DS is kindly acknowledged.

## Conflict of Interest

The authors declare that the research was conducted in the absence of any commercial or financial relationships that could be construed as a potential conflict of interest.
